# Acceptability of the Transitional Wearable Companion “+*me*” in Children With Autism Spectrum Disorder: A Comparative Pilot Study

**DOI:** 10.3389/fpsyg.2020.00951

**Published:** 2020-05-28

**Authors:** Valerio Sperati, Beste Özcan, Laura Romano, Tania Moretta, Simone Scaffaro, Noemi Faedda, Giada Turturo, Francesca Fioriello, Simone Pelosi, Federica Giovannone, Carla Sogos, Vincenzo Guidetti, Gianluca Baldassarre

**Affiliations:** ^1^Institute of Cognitive Sciences and Technologies, ISTC-CNR, Rome, Italy; ^2^Department of General Psychology, University of Padua, Padua, Italy; ^3^Italian Neurotraumatological Institute, INI-Villa Dante Division, Rome, Italy; ^4^Section of Child and Adolescent Neuropsychiatry, Department of Human Neuroscience, University of Rome Sapienza, Rome, Italy

**Keywords:** Autism Spectrum Disorder, robotics, transitional wearable companion, +*me*, early treatment, intrinsic motivations

## Abstract

+*me* is an experimental interactive soft toy, looking like a panda, developed for young children. When touched on the paws or head (inputs), the toy can emit attractive responses such as colored lights and amusing sounds (outputs). +*me* is wirelessly connected to a control tablet through which an adult caregiver can modify its input-output contingencies so as to produce different, rewarding response patterns using the same device. Given these features, we propose +*me* as a potential novel tool to support the therapy of Autism Spectrum Disorder (ASD). The allure of the device could be exploited to capture the attention and encourage the social interaction of toddlers during play activities with therapists. In this pilot study, +*me* was tested on two small groups of children aged 30–48 months, one group diagnosed with ASD and the second with Communication Disorder, a condition that often presents—especially at an early age—overlapping symptoms with ASD. The proposed play activities aimed to foster simple imitative behaviors and stimulate the engagement of the children. The results were compared with those of a previous test run on Typically Developed children. Preliminary observations, based on the analysis of video recordings, suggest that, on average, +*me* is able to encourage a positive engagement and that different groups tend to manifest some different behaviors.

## 1. Introduction

Autism Spectrum Disorder (ASD) is a Neurodevelopmental Disorder (ND), typically evident from early childhood, mainly characterized by important life-long impairments in the social and communicative areas. Although symptoms can be very heterogeneous and can range from mild to severe and there is often co-occurrence with other conditions, ASD individuals generally share difficulties in both social and emotional interactions, presenting altered or impaired communication abilities (both verbal and non-verbal), usually in the presence of restricted areas of interest and repetitive behaviors (American Psychiatric Association, 2013).

Recent reviews on ASD epidemiology show that a significant part of the population in developed countries is diagnosed with this condition: estimates range from 1.5% (≈ 1 in 67, Lyall et al., 2017) to 0.76% (≈ 1 in 132, Baxter et al., 2015). The considerable variability across statistics can be reasonably attributed to several concurrent factors, such as different methodological approaches across countries (Fombonne, 2009), increasing awareness of the condition (Elsabbagh et al., 2012), and broadening of diagnostic criteria (Lord and Bishop, 2015).

The onset of symptoms typically occurs by the age of 3 years, although they may not fully manifest until school age (Lyall et al., 2017), while the average age of a child receiving a formal ASD diagnosis is around the age of 5 years (Shattuck et al., 2009; Jónsdóttir et al., 2011; Zuckerman et al., 2015; Neimy et al., 2017). This notwithstanding, recent studies suggest that behavioral warning signs, such as atypical orienting to people, reduced eye contact, lack of response to name, and lack of social or emotional reciprocity—all pivotal precursors of the complex cognitive construct of *Social Cognition* (Happé and Frith, 2014; Pino et al., 2017)—can already emerge within the first 2 years of life (Ozonoff et al., 2010; Zwaigenbaum et al., 2013; Jones et al., 2014).

Early diagnosis is crucial. In this respect, several studies show how, on average, early rehabilitative interventions appear to be effective for toddlers with ASD (Bryson et al., 2003; Dawson et al., 2010; Reichow, 2012; Dawson, 2013; Neimy et al., 2017), both improving functional behavior and reducing the overall severity of the condition (Rogers, 1998). The effectiveness of early treatment is probably due to brain plasticity, namely the capacity of the central nervous system to modify both its function and structure in response to experience (Dawson, 2008; Calderoni et al., 2016; Izadi-Najafabadi et al., 2019), which presents maximal responsiveness in the critical period of childhood (Inguaggiato et al., 2017). Early treatment can indeed positively influence the development of important neural pathways within the social brain circuitry, especially if initiated before the full onset of core ASD symptoms (Webb et al., 2014).

One of the most promising rehabilitative approaches for early intervention exploits interactive technologies (Boucenna et al., 2014; Virnes et al., 2015), which proved to stir engagement and emotional participation in ASD subjects (Di Mascio et al., 2020). In particular, robots (Begum et al., 2016; Pennisi et al., 2016) and mechatronic toys (Mikołajewski et al., 2017) appear to be notably effective as support tools to improve the social skills of young ASD children, although most of the studies are still exploratory and are characterized by methodological limitations (Diehl et al., 2012, 2014). The efficacy of such instruments appears to rest on the clear attraction that most ASD children show toward mechanical and technological devices (Kumazaki et al., 2019), generating a high degree of motivation and engagement (Scassellati, 2007). Robots can be perceived as a sort of “playmate,” so that ASD children express an interest in socially interacting with such companions (Feil-Seifer and Matarić, 2009). It has been hypothesized that ASD children find social interactions with robots easier, as these are more predictable and thus less confusing and distressing than humans (Scassellati et al., 2012; Pennazio, 2017).

**Figure 1:**
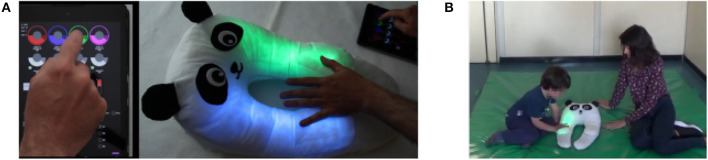
**(A)** +*me* detects the user's touch on the upper paws and responds by lighting them up, while colors are selected through the tablet. **(B)** The experimental setup: +*me* is put on the floor between the child and the researcher. The second researcher –holding the tablet– and the therapist are in the same room but do not participate to the activity.

+*me* is an experimental toy (described in section 2.1) that can be perceived by toddlers as an interactive companion. The toy is a soft panda capable of emitting several attractive responses, such as glowing colored lights and amusing sounds, when touched on the paws or caressed on the head (see Figure 1). Wearability—the panda can be worn around the user's neck—and softness are thought to arouse emotional attachment and reassuring feelings, typical features of *transitional objects* (Winnicott, 1953; Elias et al., 2011); interactive behavior aims to stimulate the curiosity and the engagement of children, relying on the intrinsic motivational drive to explore novel stimuli (Deci and Ryan, 2010; Baldassarre and Mirolli, 2013). Due to these properties, different from those of typical “rigid” robots, we refer to +*me* as a *Transitional Wearable Companion, a TWC* (see Özcan et al., 2016, for further details on the TWC concept).

This report describes a pilot experiment where +*me* was tested with two small groups of children diagnosed with ASD and with Communication Disorder (CD), a ND characterized by persistent difficulties in the acquisition and use of language (American Psychiatric Association, 2013) that frequently presents overlapping symptoms with ASD, especially at an early age (Webb et al., 2014). The main goal of the work is to evaluate the general acceptability of +*me* as an attractive toy for children with ASD in view of its potential use as a tool to support therapeutic activities. The paper also reports the results from a previous comparable pilot test run with Typically Developed (TD) children (Sperati et al., 2019) so as to assess behavioral differences between the three groups.

The rest of the paper is organized as follows. Section 2 describes the features of +*me* and the experimental protocol. Section 3 reports both quantitative and qualitative observations from the experimental sessions. Finally, Section 4 discusses the potential use of +*me* in the therapy of ASD.

## 2. Methods

### 2.1. The +*me* Device: Features and Functions

+*me* is realized with cotton fabric and soft padding (see Figure 1A) that embeds the inner Arduino-based electronics^1^. Through hidden capacitive sensors sewn under the cotton (four sensors on the paws and three on the head), +*me* can detect the user's hand contact. In response to touches (i.e., the *inputs*) it can emit colored lights and brief amusing sounds (i.e., the *outputs*, respectively through four LED strips in the paws and two speakers in the head). Through a control tablet, wirelessly connected to the device, an adult can select particular operating modes called *functions*, which modify the *inputs-output* contingencies^2^. The toy is endowed with seven functions (*F*) as follows:

- *F*_0_*:* this is the basic functioning of +*me*. The adult can select which paws are responsive to touch—all paws can be activated independently of each other—and which colors and sounds are emitted in case of contact. The available colors are red, green, blue, and magenta. The available sounds are included in a library of mp3 files.
- *F*_1_*:* each paw emits a different output if touched: a brief red light on the lower left paw; an extended blue light on the upper left paw; a brief sound (harp notes) on the upper right paw; an extended blue light plus a brief sound (spring noise) on the lower right paw.
- *F*_2_*:* a blinking red light is displayed on a random paw; if it is touched, a sound is emitted (trumpet notes) and the color turns to green. After a couple of seconds, the game restarts with another random paw.
- *F*_3_*:* if +*me* head is correctly caressed (from the left to the right ear), the panda emits a global light pattern (all paws light up with different colors) and brief music (chime notes).
- *F*_4_*:* relaxing music is played while a restful light pattern is emitted (paws light up in blue, one after the other, continuously).
- *F*_5_*:* if the upper paws are touched together, they light up in green and a brief sound is emitted (electronic ding).
- *F*_6_*:* the adult, hitting a visible button in the app, can trigger a global pattern formed by sounds (guitar notes) and mixed colors on all paws (four patterns available).

Hereinafter, we refer to the various +*me* responses as the *rewarding outcomes*, since TD children generally show enjoyment when they receive this type of feedback (Sperati et al., 2019). The rationale behind the functioning of +*me* was to build an interactive toy with several different behaviors usable for stimulating children's curiosity. Moreover, the control of (+*me*ś) behavior is *shared* between the child, who handles the panda and triggers the outcomes, and an adult, who selects the device function. This allows, at least in principle, social play activities to be set up where the child has to cooperate with the adult to obtain a desired outcome.

### 2.2. Experimental Protocol

In order to start evaluating the behavior of autistic children during social play involving +*me* and an adult, we tested *N* = 7 participants (average age 40.3±5.8 months) diagnosed with ASD [five identified as high-functioning and two as low-functioning through a cognitive evaluation by *Leiter-R scale* (Roid and Miller, 1997) or *Griffiths Mental Development Scales-II* (Griffiths, 1970), with a cut-off of 85; one was indicated to have a high level of symptoms, three to have a moderate level, and three to have a low level by *ADOS-2 module 1* (Lord et al., 2012)] and also *N* = 7 participants (average age: 39.9±6.0 months) diagnosed with CD (three with Speech Sound Disorder and four with Language Disorder). The diagnoses, based on *DSM-5* criteria (American Psychiatric Association, 2013), were made at the Department of Human Neuroscience, Section of Child and Adolescent Neuropsychiatry, University of Rome “Sapienza,” after a complete neuropsychological and neuropsychomotor assessment according to international guidelines. All participants were recruited and tested in the same department and were not subjected to any treatments. The CD group was included for comparison, as language impairments are often a common reason for ASD assessment, although the *DSM-5* does not require them (Richard et al., 2019). The experimental sessions took place in an observation room set up specifically for this purpose, where most distracting elements (e.g., pens and notebooks, baskets, toys, and drawings on walls) were removed or hidden. Each child was tested individually in the presence of three persons: the researcher who played with the participant (henceforth the *caregiver*), a second researcher in charge of recording the session with a camera and of controlling the tablet (henceforth the *controller*), and a familiar therapist. The last two persons did not take active part in the experimentation and remained silent in a corner of the room; the presence of the therapist was necessary to create a reassuring situation for the child.

Both experimental setup and procedure were, with some minor modifications, the same as a previous pilot test run on TD children (Sperati et al., 2019). The test started with a brief familiarization period lasting between 2 and 5 min during which the child was taken into the room and invited by the caregiver to play on the floor with a common toy (generally a little car or a doll). When the child felt comfortable, the +*me* (previously out of sight) was introduced and put between them, while the familiar toy was removed and hidden.

This marked the beginning of the session, lasting ~10 min; the researcher proposed to the child six play activities focused on +*me*, run in succession, each one lasting about 80/100 s. Each activity, exploiting a specific device function *F*, aimed to capture the child's attention and to stimulate the interaction with the device and the experimenter^3^. The control tablet, unless otherwise specified, was always out of sight, held by the controller, who was also in charge of selecting the proper function. The specific activities (*A*) are here described in detail:

- *A*_1_ *(one hand imitation):* the controller selects function *F*_0_ on the tablet and disables three paws; then, the caregiver touches the only responsive paw, which produces a green light and the sound of a cuckoo clock. The action is repeated three times and is accompanied by encouraging expressions like “Look here!,” “What is going on here?” “Ready, steady, go!” Then the caregiver points the +*me* at the child, who is left free to interact with it (the same procedure is repeated for all activities).
- *A*_2_ *(two hands imitation):* the controller selects function *F*_5_; then, the caregiver touches the upper paws and triggers the rewarding outcome.
- *A*_3_ *(gesture imitation):* the controller selects function *F*_3_; then, the caregiver caresses the panda head and triggers the rewarding outcome.
- *A*_4_ *(reward game):* the controller selects function *F*_2_; then, the caregiver touches the random red-blinking paw and triggers the rewarding outcome.
- *A*_5_ *(reward patterns):* the controller hands the tablet to the caregiver, who selects function *F*_6_, and then she triggers one of the available rewarding outcomes. While doing this, she highlights her gesture of touching the tablet by saying, “Look here!.” This is the only activity where the control role of the tablet is shown to the child.
- *A*_6_ *(wearability):* the controller selects function *F*_4_; then, the caregiver wears +*me* around her neck and proposes that the child wear the device.

The whole experimental session was captured with a camera, and later analyzed. Through video-editing software allowing frame-by-frame inspection, a researcher rated each clip to extract both durations (in seconds) and frequencies of the following 12 behavioral indexes:

- *touchP:* child touches +*me* (every contact between hand and device is considered for the scoring)
- *holdP:* the child holds +*me* (e.g., the child picks it up, hugs it, or flips it)
- *watchP:* the child looks at +*me*
- *refuseP:* the child refuses +*me* (the child shows aversion, irritation, or discomfort)
- *move away:* the child moves away from +*me* and the caregiver (the child loses interest or gets distracted)
- *smileP:* the child smiles at +*me*
- *smileEx:* the child smiles at the caregiver
- *cry:* the child cries
- *touchEx:* the child touches the caregiver
- *watchEx:* the child looks at the caregiver
- *pointing:* the child performs a pointing behavior with his/her hand (to +*me* or to the caregiver)
- *watchTablet:* the child looks at the tablet (only for activity *A*_5_).

This set of indexes was chosen because it roughly furnishes a quantitative description of the interactions involving the system +*me-child-caregiver*. To assess the reliability of the rating, a second researcher rated five randomly selected videos (out of 14) using the same scoring procedure. The Inter-Rater Reliability (IRR) of coders was assessed using a two-way mixed, consistency, single measures units Intra-Class Correlation (ICC, McGraw and Wong, 1996). The IRR confirmed excellent agreement between coders, being *ICC* = 0.96 and *ICC* = 0.85, respectively, for frequencies and durations (Cicchetti, 1994; Hallgren, 2012). The data from the first coder were then used for the subsequent results analysis.

This study was approved by the *Ethical Committee* of *ISTC-CNR*; parents were informed about the purpose of the research and gave written consent to the experimentation, in accordance with the principles established in the *Declaration of Helsinki* (World Medical Association, 2001).

## 3. Results

**Figure 2:**
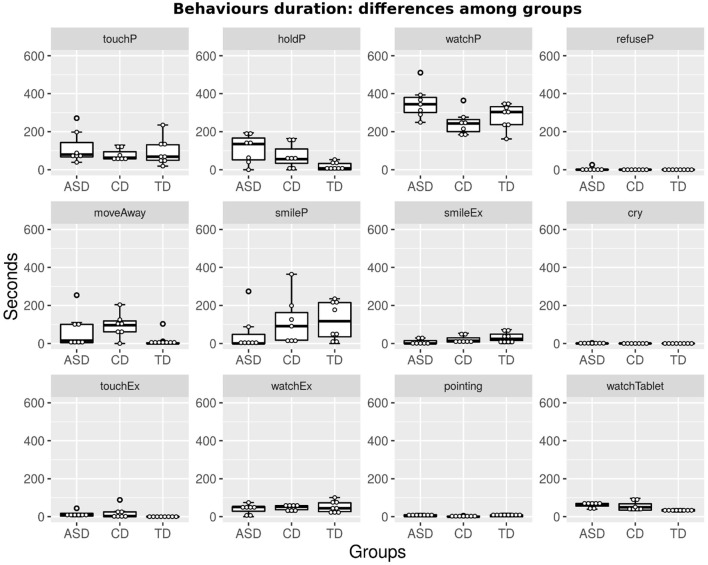
Each graph shows boxplots for the ASD, CD, and TD groups of the duration in seconds of a behavioral index, measured during the whole session.

**Figure 3:**
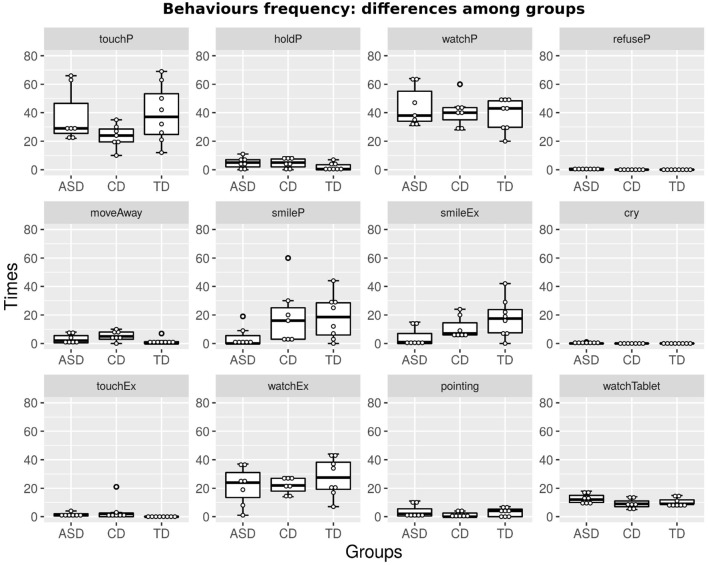
Each graph shows boxplots for the ASD, CD, and TD groups of the frequency of a behavioral index, measured during the whole session.

Figures 2, 3 show boxplots of the duration and frequency of the 12 behavioral indexes in the ASD and CD groups^4^. The plots in the figures also show data from the previous pilot experiment (Sperati et al., 2019) run with TD children (*N* = 8, average age 30.2±2.8 months^5^). Visual comparison of the boxplots related to the ASD, CD, and TD children (henceforth simply called ASDs, CDs, and TDs) allows the evaluation of the main differences among the three groups. We also report qualitative observations^6^, which are useful for interpreting the data.

In general, all children spend a certain amount of time exploring +*me* (see *touchP* and *watchP* indexes in both figures). Participants tend to imitate the caregiver, paying attention to her gestures; for example, in activities *A*_1_ and *A*_2_, they mostly handle +*me* paws, producing lights and sounds, and in *A*_3_, they tend to caress the panda head, as shown by the caregiver. These results seem to suggest a potential role for +*me* in facilitating dyadic (e.g., imitation) and triadic (e.g., joint attention) behaviors involving the child, the toy, and the caregiver (Clifford and Dissanayake, 2009).

Compared with TDs, ASDs and CDs have a higher tendency to occasionally move away from the setting (see index *moveAway*) and to move around within the room (some of the ASDs explore the room in detail). This behavior does not necessarily mean “loss of interest,” as it is often observed along with index *holdP*: some of ASDs move away from the shared play area while keeping the pillow and putting it in their personal space. These results highlight some important differences between TDs and both ASDs and CDs, suggesting that, in ASD and CD, the use of personal space may be a potential transdiagnostic feature shared across different NDs.

As expected, ASDs exhibit a decreased emotional involvement during the various activities, as shown by a lack of smiling responses both to +*me* and to the caregiver (see, respectively, *smileP* and *SmileEx* in both figures).

Activity *A*_4_ requires that the participant “understands” the game; interestingly, TDs and CDs correctly perform the play (they chase the red-blinking paw with their hand), while five out of seven ASDs tend to touch the panda paws, repeating the gestures done in previous activities. Findings on ASDs suggested a role for +*me* in highlighting not only a potential transdiagnostic feature shared across different NDs but also specific potential behavioral features characterizing ASD that may be useful in supporting traditional diagnosis from the first years of life. Further studies are necessary to support these preliminary findings and their potential role in discriminating ASDs from children with other NDs.

**Figure 4:**
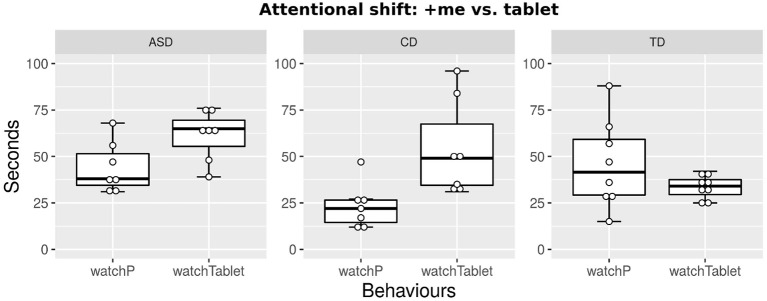
In activity *A*_5_, although the tablet is an attention-capturing stimulus, the participants do not completely ignore +*me* and continue to look at it when producing a rewarding outcome.

As expected, during activity *A*_5_, the tablet immediately captures the attention of all participants, who try to touch it. We note, however, that the shift of attention is not total, as children continue to look at +*me* when the rewarding outcome is produced (see Figure 4).

Finally, activity *A*_6_ aimed to evaluate the *wearability* of +*me* : as expected, children from all groups tend to remove it from their neck, but some of them try to sporadically wear it again, confirming that the panda shape can encourage this behavior. This feature was designed for older children and relies on the observation that tight clothing can often exert a reassuring effect on ASDs (Mullen et al., 2008; Stephenson and Carter, 2009).

We are aware that the aforementioned observations have to be considered as preliminary results, though they seem to encourage the use of +*me* as a tool to support social play. For a better understanding of the interactions between child and caregiver mediated by the toy, it is our opinion that the temporal sequences of behaviors (e.g., looking at the pattern of the child's gaze during the caregiver's requests) are a very important point to be analyzed in future experiments.

## 4. Discussion

ASD is a neurodevelopmental disorder imposing significant social and financial burdens (Lyall et al., 2017). Rehabilitative treatments within early childhood have proved to be effective in ameliorating the severity of the condition through the reinforcement of social competencies (Volkmar et al., 2014). Nevertheless, such interventions can be, by definition, complex: ASD children often present a lack of interest toward other people and experience difficulties with responding to social interactions and with engaging in meaningful play activities (Godin et al., 2019). Thus, any supporting tool possibly useful to stimulate and increase social engagement of such patients is worth investigating.

+*me* is an interactive toy designed for this purpose (Özcan et al., 2016). The toy aims to increase the attractiveness of *shared play*, an arguably important goal for ASD interventions (see the “Playfulness” concept in Godin et al., 2019). In this pilot study, two small groups of children with ASD and CD were observed during short play activities involving the device and an adult caregiver. The experimental protocol, used in a previous pilot on TD children (Sperati et al., 2019), aimed to evaluate the general acceptability of +*me* as a toy able to arouse curiosity, encourage interaction, and elicit simple social behaviors. Our observations suggest positive responsiveness by ASDs and CDs toward +*me* and the caregiver in terms of attention and engagement (with ASDs showing less emotional involvement), even if less intense when compared to TDs.

Although the proposed activities did not serve any specific therapeutic purpose, the interactive features of +*me* contributed to eliciting basic social responses in the participants (e.g., imitative behaviors and joint attention) and to maintaining the attentional focus on the tasks shared with the caregiver. These results are consistent with the observation that social play activities with immediate visual and auditory feedback characterized by consistency, predictability, and physical contingency (elements also featured by +*me*) elicit a high degree of response in ASD children (Vernon et al., 2012).

Methodological limitations inherent to the exploratory nature of the study must be taken into account in the evaluation of results. Firstly, the current analysis relies on small samples of participants; thus, future experiments should test larger groups to confirm the preliminary observations presented here. Secondly, to assess the efficacy of +*me* a control condition using a comparable toy lacking the features of +*me* should be tested.

Relying on these preliminary encouraging results, we propose +*me* as a versatile supporting tool to help therapists to set-up interactive play activities for early treatment with ASDs. For example, the features of +*me* could be exploited within the setting proposed in the Early Start Denver Model (ESDM); this is a comprehensive intervention for children aged between 12 and 48 months based on natural play and focused on building positive relationships. ESDM is currently one of the most effective evidence-based treatments, demonstrating significant gains in the overall developmental quotients of social, language, and cognitive skills (Dawson et al., 2010; Vivanti and Dissanayake, 2016; Colombi et al., 2018).

Finally, taking into consideration both the very young age of participants and the different behaviors between groups, we envisage another potential use of the device, worthy of further investigation: as a means for differential diagnosis. Especially at an early age, ASD and CD symptoms often overlap (Webb et al., 2014), so that a language impairment or delay, although not required for a diagnosis of ASD under *DSM-5*, is one of the most common reasons for ASD evaluation, despite about 7% of the general population being diagnosed with CD without ASD (Richard et al., 2019).

## Data Availability Statement

The raw data (participants scoring grids), supporting the conclusions of this article will be made available by the authors, without undue reservation, to any qualified researcher.

## Ethics Statement

This study was approved by the Ethical Committee of ISTC-CNR; parents were informed about the purpose of the research and gave written consent to the experimentation, in accordance with the principles established in the Declaration of Helsinki (World Medical Association, 2001).

## Author Contributions

The manuscript was written by VS, who also developed the hardware and control software of the device, +*me* was designed by BÖ, who assembled the current prototype and also developed the original concept of a *Transitional Wearable Companion*, the experiment with children was run by LR, GT, SS, and SP, data analysis was performed by VS, LR, TM, NF, and FF, the experimental design was conceived by TM, SS, LR, and GB, the experiment was supervised by GB (for ISTC-CNR) and VG, CS, and FG (for University of Rome Sapienza), the manuscript was revised by all authors.

The supplementary images display magnifications of the data shown in Figures 2, 3.

## Conflict of Interest

The authors declare that the research was conducted in the absence of any commercial or financial relationships that could be construed as a potential conflict of interest.

## Footnotes

^1^www.arduino.cc

^2^See https://vimeo.com/259130096 for video material.

^3^Function *F*_1_ was not used.

^4^Magnified boxplots can be seen in the Supplementary Material.

^5^The data shown are for the participants in the “older group” in the cited article.

^6^See https://drive.google.com/open?id=1dVRLPCwbuyko0z02k0w3KJN3dgsTCMRb for supporting video material.
