# Supplementary material for: Acceptability of the Transitional Wearable Companion “+me” in Children With Autism Spectrum Disorder: A Comparative Pilot Study
Source: Front Psychol. 2020 May 28;11:951. doi: 10.3389/fpsyg.2020.00951 (PMC7270338; doi:10.3389/fpsyg.2020.00951)
Supplement: Supplementary file 1 [file Data_Sheet_1.PDF]

Supplementary Material

1 SUPPLEMENTARY TABLES AND FIGURES

Data relating to Figures 2 and 3 of the main articleare are displayed in pairs (*ASD vs TD*, *ASD vs CD*, *CD vs TD*) and in different scales, to better appreciate the differences among groups.

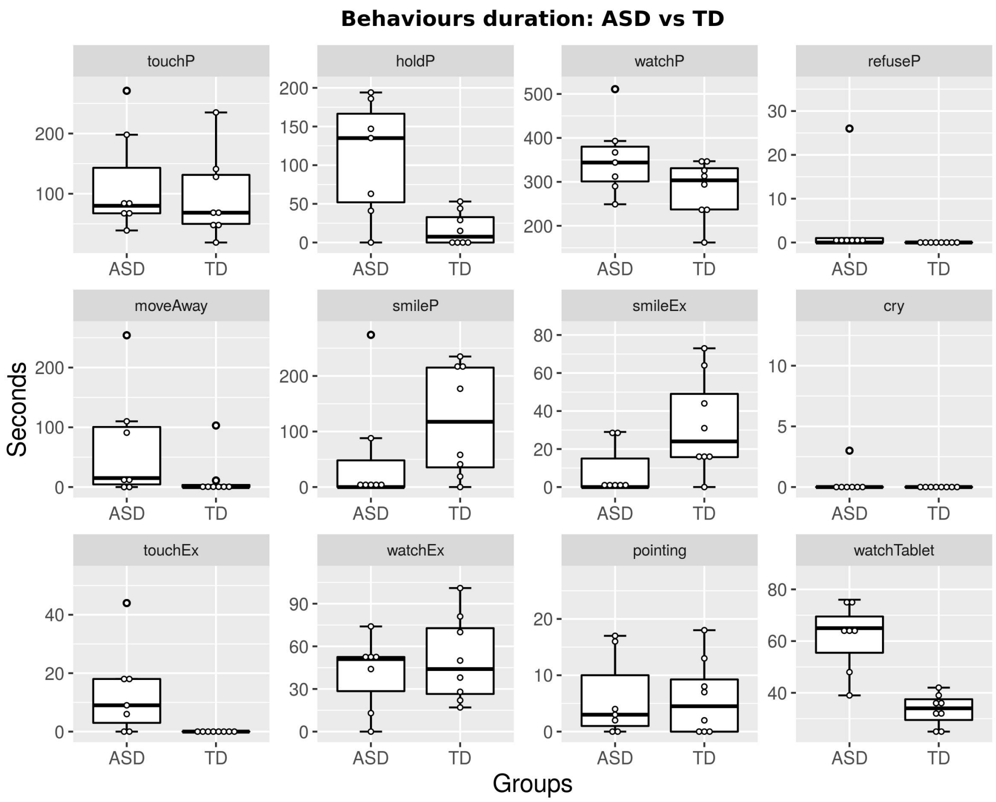

Figure S1. ASD vs TD durations.

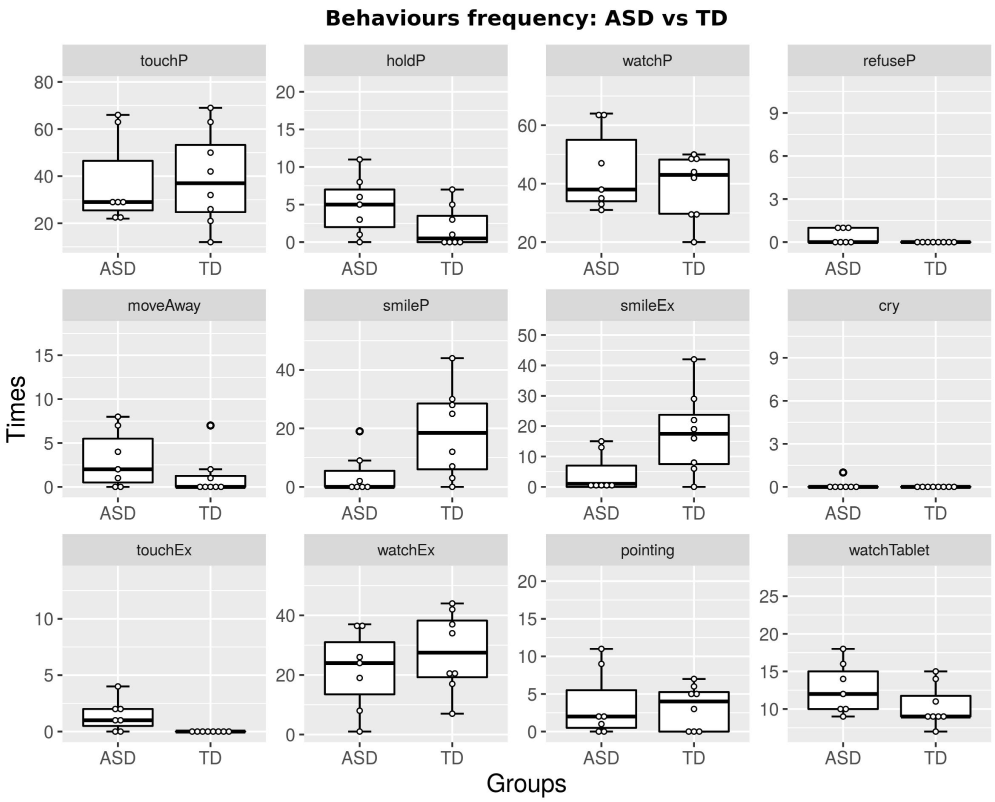

Figure S2. ASD vs TD frequencies.

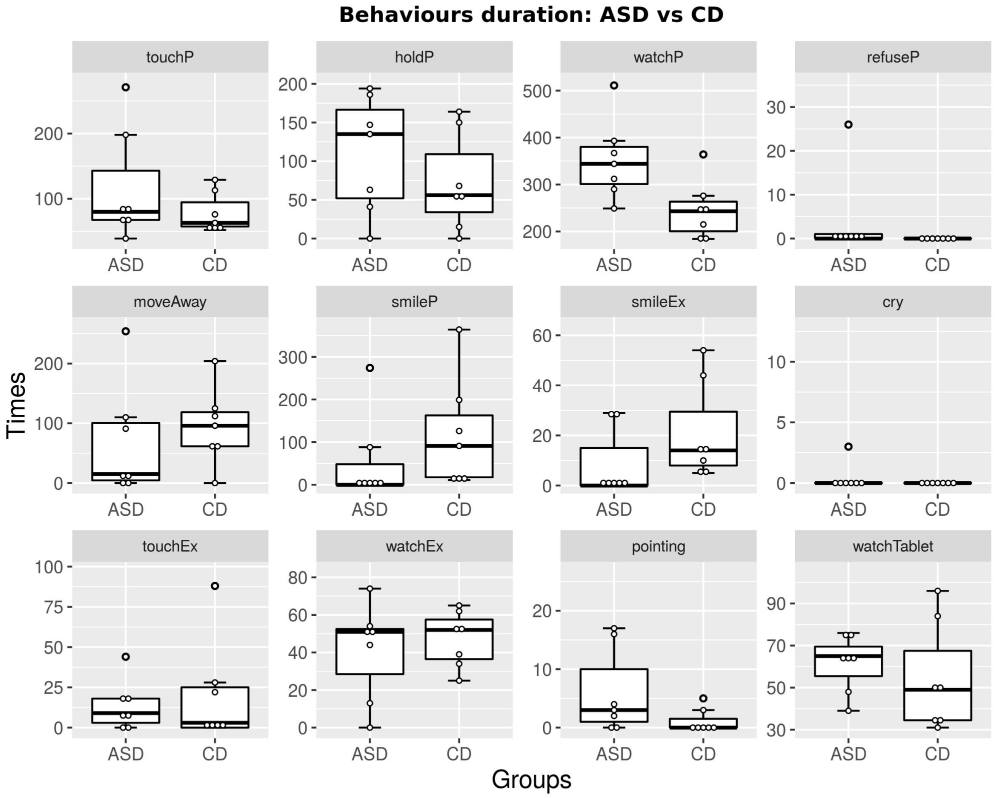

Figure S3. ASD vs CD durations.

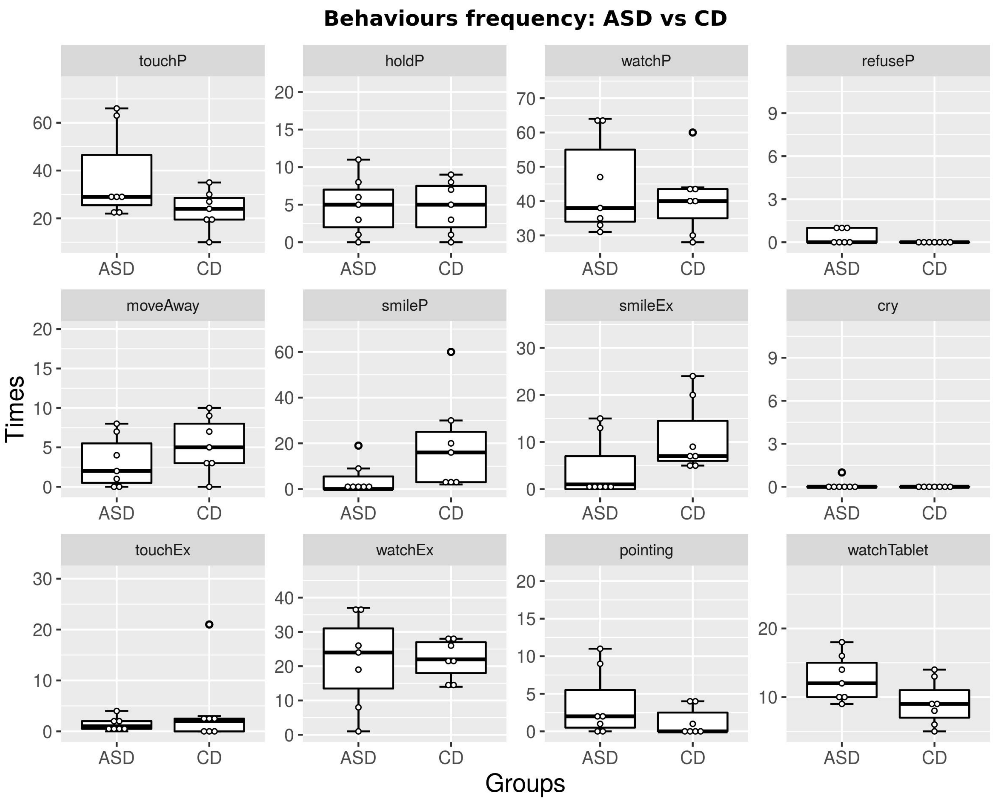

Figure S4. ASD vs CD frequencies.

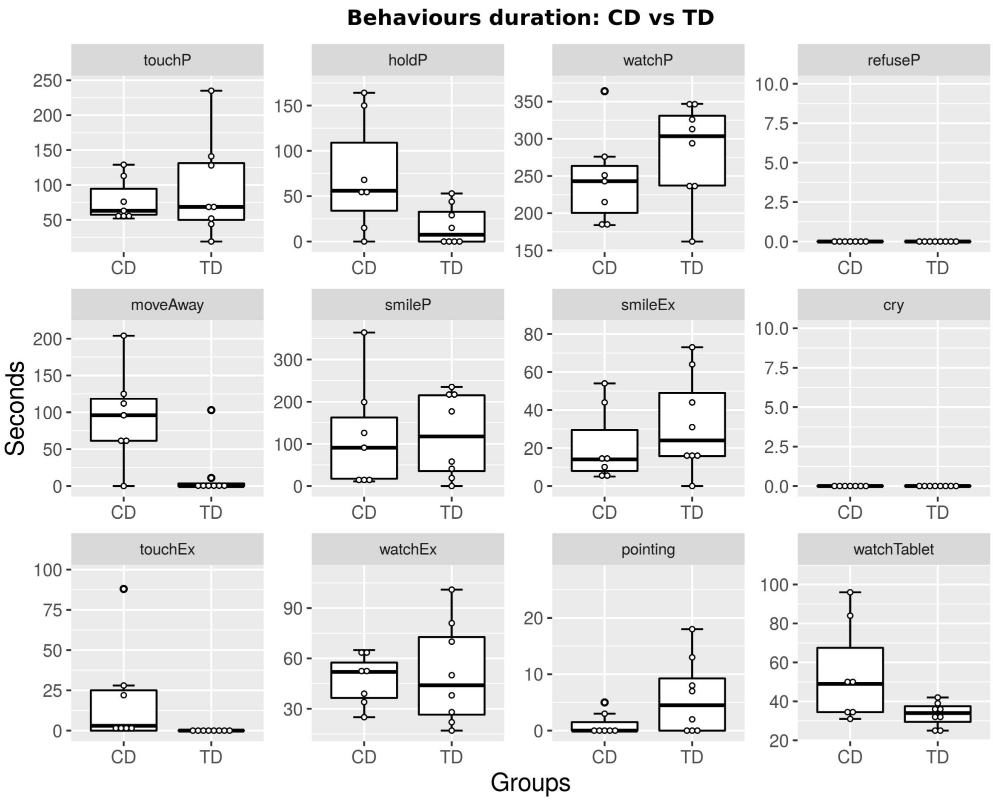

Figure S5. CD vs TD durations.

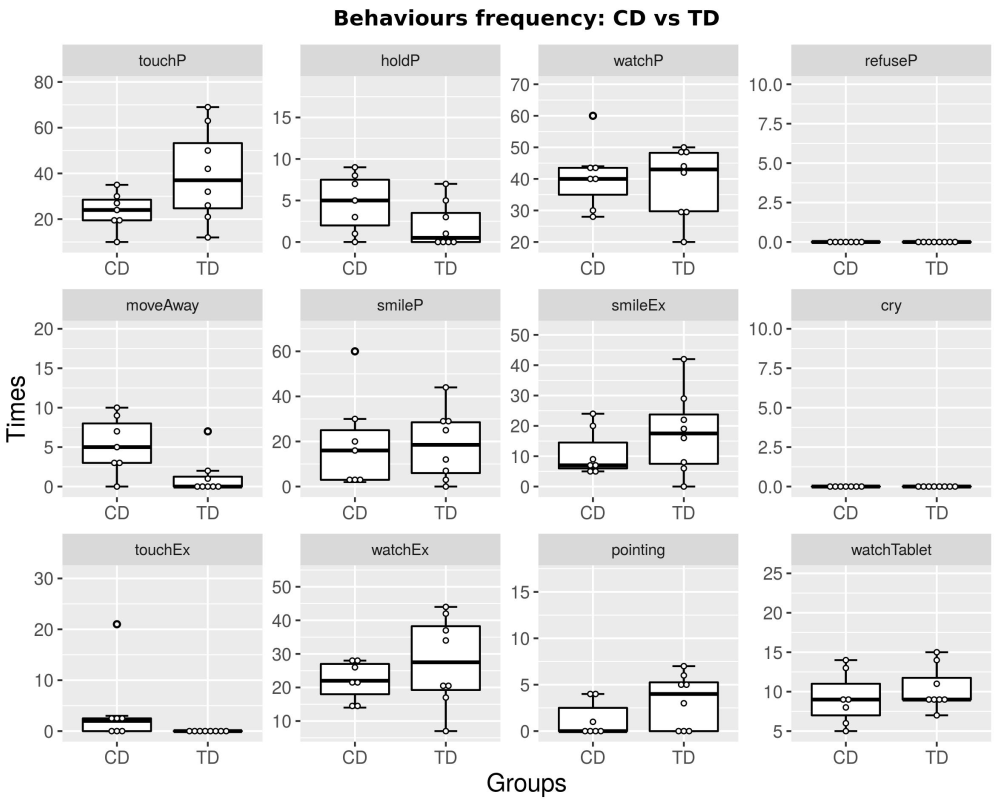

Figure S6. CD vs TD frequencies.
